# Single-cell transcriptomic analysis of hematopoietic progenitor cells from patients with systemic lupus erythematosus reveals interferon-inducible reprogramming in early progenitors

**DOI:** 10.3389/fimmu.2024.1383358

**Published:** 2024-05-08

**Authors:** Anastasia Filia, Ioannis Mitroulis, Catherine Loukogiannaki, Maria Grigoriou, Aggelos Banos, George Sentis, Stavroula Giannouli, Vassiliki Karali, Emmanouil Athanasiadis, Ioannis Kokkinopoulos, Dimitrios T. Boumpas

**Affiliations:** ^1^ Laboratory of Autoimmunity and Inflammation, Center of Clinical, Experimental Surgery and Translational Research, Biomedical Research Foundation Academy of Athens, Athens, Greece; ^2^ 1st Department of Internal Medicine, University Hospital of Alexandroupolis, Democritus University of Thrace, Alexandroupolis, Greece; ^3^ 2nd Department of Internal Medicine, Ippokrateion Hospital, National and Kapodistrian University of Athens, Athens, Greece; ^4^ 4th Department of Internal Medicine, Attikon University Hospital, National and Kapodistrian University of Athens Medical School, Athens, Greece; ^5^ Medical Image and Signal Processing Laboratory, Department of Biomedical Engineering, University of West Attica, Athens, Greece

**Keywords:** single cell RNA sequencing, SLE, bone marrow, hematopoiesis, interferon signaling

## Abstract

**Introduction:**

Immune cells that contribute to the pathogenesis of systemic lupus erythematosus (SLE) derive from adult hematopoietic stem and progenitor cells (HSPCs) within the bone marrow (BM). For this reason, we reasoned that fundamental abnormalities in SLE can be traced to a BM-derived HSPC inflammatory signature.

**Methods:**

BM samples from four SLE patients, six healthy controls, and two umbilical cord blood (CB) samples were used. CD34+ cells were isolated from BM and CB samples, and single-cell RNA-sequencing was performed.

**Results:**

A total of 426 cells and 24,473 genes were used in the analysis. Clustering analysis resulted in seven distinct clusters of cell types. Mutually exclusive markers, which were characteristic of each cell type, were identified. We identified three HSPC subpopulations, one of which consisted of proliferating cells (*MKI67* expressing cells), one T-like, one B-like, and two myeloid-like progenitor subpopulations. Differential expression analysis revealed i) cell cycle-associated signatures, in healthy BM of HSPC clusters 3 and 4 when compared with CB, and ii) interferon (IFN) signatures in SLE BM of HSPC clusters 3 and 4 and myeloid-like progenitor cluster 5 when compared with healthy controls. The IFN signature in SLE appeared to be deregulated following TF regulatory network analysis and differential alternative splicing analysis between SLE and healthy controls in HSPC subpopulations.

**Discussion:**

This study revealed both quantitative—as evidenced by decreased numbers of non-proliferating early progenitors—and qualitative differences—characterized by an IFN signature in SLE, which is known to drive loss of function and depletion of HSPCs. Chronic IFN exposure affects early hematopoietic progenitors in SLE, which may account for the immune aberrancies and the cytopenias in SLE.

## 1 Introduction

Hematopoietic stem and progenitor cells (HSPCs) represent a primitive multipotent population that gives rise to all blood cell types (1). HSPCs reside in the bone marrow (BM) niche and remain in a quiescent state. Under hematopoietic stress, including inflammation, these cells respond with proliferation and differentiation, in order to replenish any progeny needed (2, 3).

HSPCs are an integral part of the immune response with the ability to sense inflammatory stimuli in infectious and chronic inflammatory diseases, since they are equipped with receptors for pathogen-derived stimuli, several cytokines, such as interferons and IL-1β, or myeloid-related growth factors. Studies in animal models have shown that HSPCs are important players in the initiation and perpetuation of the inflammatory processes in systemic lupus erythematosus (SLE) (4), rheumatoid arthritis (5), and spondyloarthritis (6).

A prolonged exposure to inflammatory stimuli during chronic inflammatory diseases has long-lasting effects on the BM cell output’s nature through epigenetic modifications in HSPCs (1, 7, 8). The reprogramming of HSPCs by inflammatory stimuli can alter mature cell output, resulting in the generation of effector cells with inflammatory properties (7, 9, 10).

It has been demonstrated that an interplay between environmental, genetic, and epigenetic factors promotes SLE (11). A key observation in SLE is that most cells participating in its pathogenesis such as lymphocytes, monocytes, and neutrophils originate from HSPCs (11). We have reasoned that the fundamental molecular aberrations in SLE may be traced back in the HSPCs within the BM. To this direction, we collected human CD34^+^ cells from healthy and SLE patients as well from newborn umbilical cord blood (CB) and we performed deep RNA sequencing analysis, at the single-cell level. We report that interferon signaling, which is a major player in disease pathogenesis, already affects early hematopoietic progenitors in SLE. Differential mRNA splicing analysis underscored alterations in the cell cycle and DNA repair machineries in SLE BM-derived HPSCs, when compared with healthy-derived HSPCs. These data suggest that HSPCs act as sensors of interferon-related inflammatory signals, initiating the inflammatory process that characterizes SLE.

## 2 Methods

### 2.1 Human samples

BM aspirates were obtained from four SLE individuals and six age- and sex-matched healthy volunteers. Patients’ clinical and serological characteristics are summarized in **Supplementary Table 1**. Informed consent was obtained from all patients prior to sample collection (Athens, Greece, protocol 10/22-6-2017). Two umbilical CB samples were donated from the Hellenic Cord Blood Bank, Biomedical Research Foundation of Academy of Athens (BRFAA).

### 2.2 Isolation of human CD34+ progenitor cells

Human heparinized BM aspirate (10 ml) was collected from healthy and SLE patients and subjected to density gradient centrifugation using Histopaque-1077 (Sigma-Aldrich). Briefly, blood was diluted 1:3 PBS and carefully layered over Histopaque medium. Tubes were centrifuged at 500 g for 30 min (no break) at room temperature. BM mononuclear cells were isolated using Histopaque-1077 (Sigma-Aldrich). BM mononuclear cells were washed, and erythrocytes were lysed with RBC buffer (420301, BioLegend). CD34^+^ cells were isolated using EasySep™ Human CD34 Positive Selection Kit II (18056, StemCell Technologies). Purity was tested and was >95%.

### 2.3 Single cell RNA sequencing data processing

Human CD34^+^ cells were loaded on Fluidigm C1 IFC. Single-cell capture and RNA extraction were performed on the IFC. Reverse transcription and amplification were performed using the SMART-Seq v.4 cDNA synthesis kit, and libraries were prepared using the Illumina Nextera XT kit. Libraries were sequenced 75 bp or 150 bp paired-end on the Illumina MiSeq or NextSeq500 platforms. Single-cell RNA-sequencing experiments were carried out at the Greek Genome Center, BRFAA.

Sequencing files were demultiplexed and converted to FASTQ format using Illumina bcl2fastq software. FASTQC v.0.11.7 software was used for quality control. Adapters and low-quality bases (Q<30) were trimmed from the 3′ end of the reads using Cutadapt v.1.18 (12). Alignment was performed against the primary assembly of GRCh38 using STAR v.2.6.1b (13), and quantification was performed using gencode_v29 annotation using HTSeq v.0.11.1 (14).

Quality control analysis was performed in order to remove poor-quality cells, which could be dead or dying. Cells were excluded if the alignment rate was <50%, the numbers of detected genes (at least one count) were <200 and >6,000, and the percentage of reads mapping to mitochondrial genes was >20%. Following filtering with the above criteria, 426 were used for further analysis.

The Seurat R package (v.4) (15) and harmony (16) were used for normalization and graph-based clustering. A resolution of 0.7 was used for UMAP clustering. The FindMarkers function was used for marker identification using the wilcox.test method (cell fraction in each subpopulation >0.2, logFC >0.2, and p < 0.01). The cell-cycle state of each cell was predicted by scoring cells for their cell cycle phase using Seurat’s CellCycleScoring function. The stemness score of each cell was calculated using the UCell R package (17) with a gene set as reported elsewhere (18). Pairwise Mann–Whitney rank-sum tests were performed for the stemness score between clusters. The interferon (IFN) score was calculated accordingly using a gene set, which is a union of modules M1.2, M3.4, and M5.12 as reported elsewhere (19).

### 2.4 Gene regulatory network analysis

The single-cell RNA-seq data were further analyzed by using SCENIC (20). SCENIC is a computational method that reconstructs gene regulatory networks and identifies cell states from single-cell RNA-seq data. There are three major R/Bioconductor packages that SCENIC depends on GENIE3 (21), RcisTarget (20), and AUCell (20). GENIE3 identifies potential transcription factor (TF) targets that are coexpressed with those TFs. Then, RcisTarget identifies the direct targets via cis-regulatory motif analysis and creates the regulons (transcription factor regulatory networks). Lastly, AUCell scores the activity of each regulon on single cells. It should be noted that all graphs except violin plots were constructed using the binary values of the AUCell algorithm. Specifically, SCENIC was utilized to create the regulons, estimate the transcription factors’ activity scores, and perform an enrichment analysis on them. Differential regulon activity analysis between SLE and healthy controls was performed in each regulon cluster. Differential regulon activity was considered significant when p < 0.05. Results were visualized by UMAPs, heatmaps, pie charts, and violin plots. Scripts were based on the work of Zhu et al. (22) and the SCENIC vignettes offered by Aibar et al. (20).

### 2.5 Differential expression

The Seurat FindMarkers function was used for differential expression analysis using the wilcox.test method between a) SLE and H controls and b) H vs. CB in each cluster. No DE was performed If cells per condition per cluster were less than 10. Gene set enrichment analysis (GSEA) (23) was performed in order to reveal enriched signatures in our gene sets based on the Molecular Signatures Database v.2022.1.Hs collections (Hallmark gene sets, GO Biological Processes, KEGG, and REACTOME pathways). Gene sets were ranked by taking the –log10 transform of the p-value multiplied by the fold change. Significantly upregulated genes were at the top and significantly downregulated genes were at the bottom of the ranked list. GSEA pre-ranked analysis was then performed using the default settings. Enrichment was considered significant when false discovery rate (FDR) < 0.05.

### 2.6 Differential alternative splicing

Differential alternative splicing (DAS) was performed between SLE and healthy controls for each cluster separately using rMATS turbo v4.1.2 (24). BAM files were merged for each participant per cluster and used as input for the detection of differential alternative splicing (DAS) events between SLE and healthy controls in each cluster. Briefly, the number of supporting reads was counted by the junction reads only. For each event, the difference in the percent-spliced-in (PSI) values, which represent the fraction of transcripts that include a particular exon or spliced site, between SLE and healthy controls was calculated. A likelihood ratio test within rMATS was performed, and DAS events between SLE and healthy controls were considered significant when FDR <0.05. Enrichment analysis was performed using the gene names showing significant DAS events using enrichR (25). Enrichment was considered significant when FDR <0.05. Sashimi plots were created to visualize the splicing patterns of genes between SLE and healthy controls using the function rmats2sashimiplot (https://github.com/Xinglab/rmats2sashimiplot#usage).

## 3 Results

### 3.1 Single-cell transcriptomes of HSPCs in CB and BM

**Figure 1:**
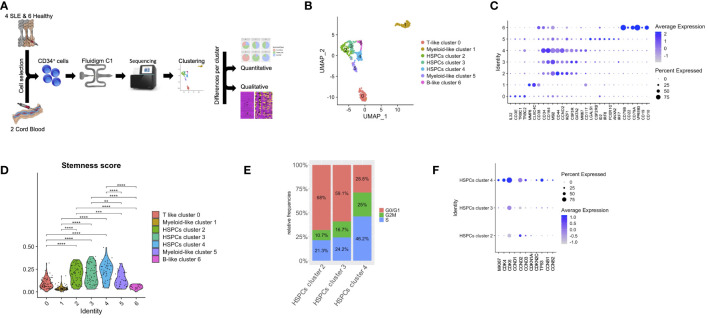
Lineage-specific alterations in HSPCs using single-cell transcriptomics in patients with SLE. **(A)** Diagram of analysis pipeline. **(B)** Uniform manifold approximation and project (UMAP) visualization of HSPC cells (CD34^+^) irrespective of disease or developmental stage (cord blood, n = 2, healthy bone marrow (BM), n = 6, SLE BM, n = 4) based on single-cell transcriptomes. Each dot represents a single cell; colors indicate cell clusters with numbered labels and cell type annotations. **(C)** Dot plot of selected marker genes in each identified cluster. **(D)** Violin plots of stemness signature score of each cluster (Mann–Whitney rank-sum test p values, **p < 0.01, ***p < 0.001, ****p < 0.0001). **(E)** Bar plot of cell-cycle phase assignments for captured cells in the HSPC clusters 2, 3, 4. **(F)** Dot plot of selected cell-cycle genes in HSPC clusters 2, 3, 4.

We sought to assess the molecular changes that take place in the HSPC signature from early life to adulthood, as well as to address the effect of SLE-dependent inflammation in this modulation at the single-cell level. To this end, single-cell RNA sequencing was performed in isolated CD34^+^ umbilical cord blood (CB) (n = 2) and bone marrow (BM) cells from healthy adult individuals (n = 6) and patients with SLE (n = 4) (**Figure 1A**). The use of CB-derived cells would be acting as a “naive” CD34+ hematopoietic progenitor cell population that has not been affected by the BM microenvironment, therefore providing a reference point for BM-derived CD34+ HPSC intrinsic differences between healthy and SLE patients. In total, we obtained transcriptomes of 426 single cells following quality control filtering (see Methods), 177 of which were healthy BM, 141 were SLE BM, and 108 were CB cells. The median number of reads was 1,259,063 (range 73,279–6,281,814). There was no statistically significant difference in the total number of reads between the three sample types (Kruskal–Wallis chi-squared = 0.33, p = 0.85, **Supplementary Figure 1**). However, there was a statistically significant difference in the total number of detected genes between the three sample types (Kruskal–Wallis chi-squared = 14.7, p < 0.001) with SLE samples expressing more genes compared with the other two sample types (**Supplementary Figure 2**).

Unsupervised clustering partitioned the cells in seven clusters (**Figure 1B**). Clusters were further defined based on the expression of lineage markers (26, 27), differentially expressed genes in each cluster compared with all others (**Figure 1C**; **Supplementary Table 2**) as well as annotation against a reference dataset (**Supplementary Figure 3**). Specifically, we identified a cluster characterized by T-cell markers (cluster 0; T-like progenitors instead of T-like prog), including *IL32*, *CD3E*, *TRBC1*, and *TRBC2*; three clusters that comprised cells expressing HSPC-related genes (cluster 2, 3, 4; HSPCs), such as *CD164*, *MSI1*, *IKFZ1*, *GATA2*, and *CD34*; two clusters characterized by the expression of myeloid-related genes (clusters 1 and 5; myeloid-like progenitors instead of myeloid-like prog), such as *MP8*, *CLEC4C* and *CD117*, *LGALS1*, *CSF2RB*, *IRF8*, *FCER1G*, and *ANXA2*; and finally a cluster characterized by B-cell markers (cluster 6; B-like progenitors instead of B-like prog), such as *CD79A*, *CD79B*, *VPREB3*, *CD22*, *CD19*, and *CD10* (**Figure 1C**). Of note, HSPC cluster 4 was characterized by the expression of the cell-cycle genes *MKI67* and *CCND2*, suggesting characterization of proliferating HSPCs (**Figure 1C**). Based on the expression of genes associated with HSPC stemness (stemness score), we were able to confirm that clusters 2, 3, and 4 included cells that were characterized by a stem cell signature (**Figure 1D**). We then focused on these three HSPC clusters and assessed the cell-cycle state of each cell, by scoring cells for their cell-cycle phase (see Methods). This analysis revealed that HSPC cluster 4 was formed by proliferating cells (**Figure 1E**). For instance, increased frequency of cells expressing *MKI67*, *CDK4*, *CDK6*, *CDKN2C*, and *TP53* and higher expression of these genes was observed in HSPCs cluster 4 (p < 0.01, **Figure 1F**).

### 3.2 Single-cell transcriptomes of HSPC subpopulations in SLE BM

**Figure 2:**
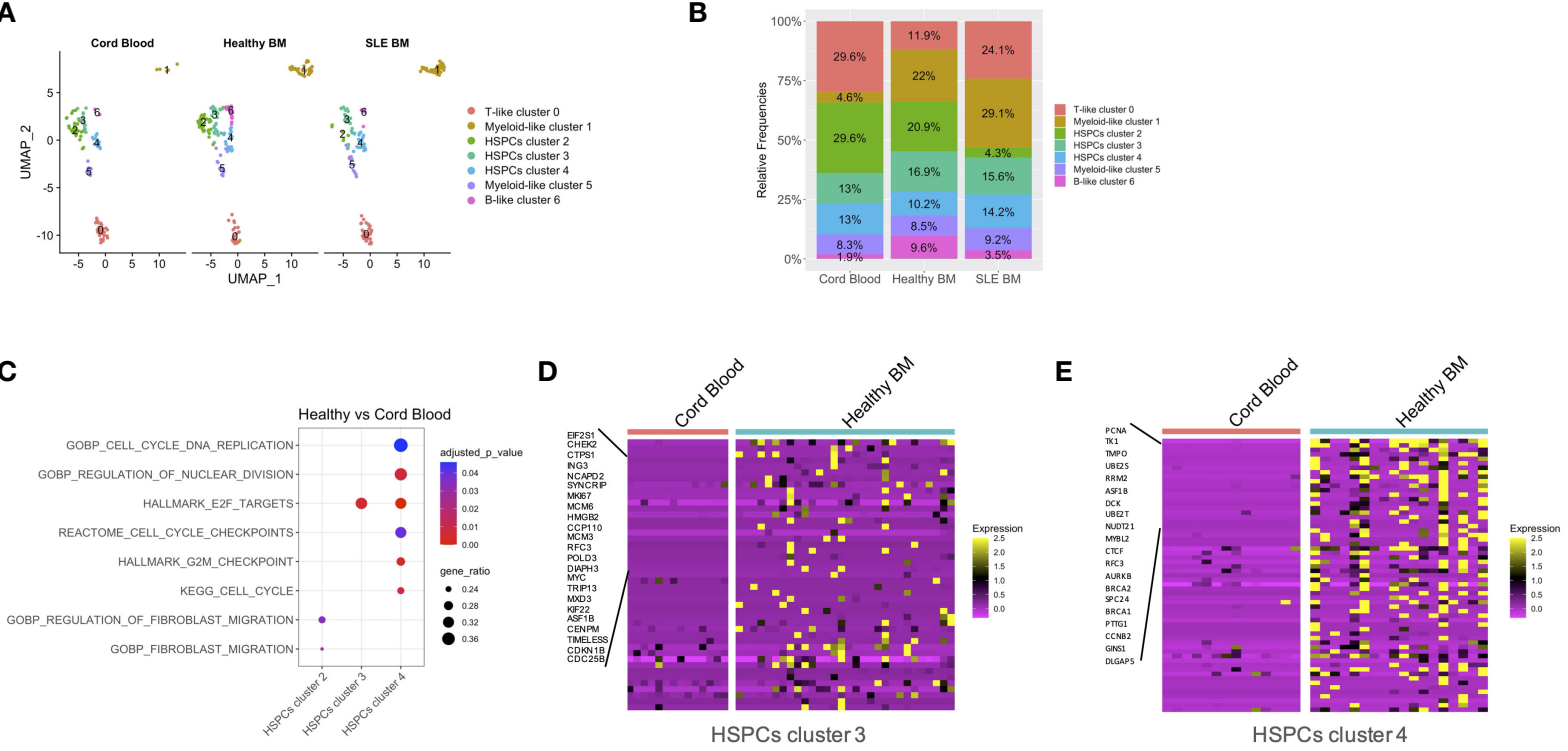
Quantitative differences per condition and qualitative differences in patients with SLE versus CB. **(A)** Uniform manifold approximation and project (UMAP) visualization of HSPC cells (CD34+) per sample type (cord blood, n = 2, healthy bone marrow (BM), n = 6, SLE BM, n = 4) based on single-cell transcriptomes. **(B)** Bar plot of cluster assignments for captured cells in each sample type. **(C)** Dot plot of GSEA-enriched terms following differential expression analysis between healthy controls and CB samples in HSPC clusters 2, 3, 4. **(D, E)** Heatmap of genes supporting the enriched cell-cycle-related pathways in healthy controls vs. CB in HSPC clusters 3 and 4.

We then studied whether SLE-dependent inflammatory signals act specifically to subpopulations of progenitor cells in the BM. To do so, we initially assessed the distribution of CB, healthy BM, and SLE BM CD34^+^ cells (**Figure 2A**). Regarding the HSPC clusters 2, 3, and 4, CD34^+^ cells from healthy adults were equally distributed to the three HSPC clusters, whereas the majority of CD34^+^ CB cells and only few SLE cells were present in HSPC cluster 2 compared with clusters 3 and 4, suggesting that this cluster corresponds to a primitive cell population (**Figures 2A, B**). Additionally, there was an underrepresentation of CD34^+^ SLE and CB cells in B-like cluster 6. Taken together, from the naive state of CB cells to HSPCs derived from the BM of control subjects and then of patients with SLE, there is an underrepresentation of the primitive cells of cluster 2. In parallel to these quantitative changes, we addressed whether there are changes in gene expression within different clusters of HSPCs using differential expression analysis. GSEA showed a positive enrichment of cell-cycle-associated signatures, including cell cycle and DNA replication, regulation of nuclear division, E2F targets, and G2M checkpoints in healthy BM of HSPCs cluster 4 (**Figures 2C, D**; **Supplementary Table 3**) and E2F targets in healthy BM of HSPCs cluster 3 when compared with CB of HSPCs cluster 4 and 3, respectively (**Figures 2C, E**; **Supplementary Table 3**).

**Figure 3:**
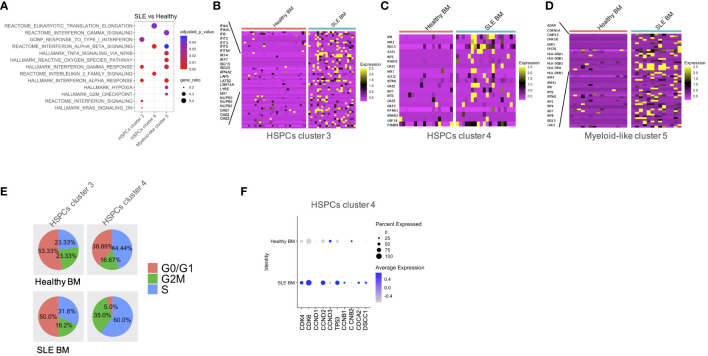
Qualitative differences in patients with SLE compared with healthy controls. **(A)** Dot plot of GSEA enriched terms following differential expression analysis between SLE and healthy controls in HSPC clusters 3 and 4 and myeloid-like cluster 5. **(B–D)** Heatmaps of genes supporting the enriched IFN-related pathways in SLE BM vs. healthy controls in HSPC clusters 3 and 4 and myeloid-like cluster 5, respectively. **(E)** Pie charts showing the composition of each cell-cycle phase in healthy BM and SLE BM in HSPC clusters 3 and 4. **(F)** Dot plot of selected cell-cycle genes in healthy BM and SLE BM in HSPC clusters 4.

GSEA between SLE and healthy cells per cluster showed a positive enrichment of interferon signatures in the SLE HSPCs cluster 3 and 4 and myeloid-like cluster 5 cells (**Figures 3A–D**; **Supplementary Table 4**; **Supplementary Figure 4**). This was accompanied by a positive enrichment of proliferation and differentiation in HSPCs, including oxidative phosphorylation and G2-M checkpoint signatures in the SLE myeloid-like cluster 5 cells (**Figure 3A**). Of note, when we compared the cell-cycle state of control and SLE HSPCs in clusters 3 and 4, we observed that the vast majority of SLE HSPCs in cluster 4 was in the S/G2-M phase (**Figure 3E**), whereas the gene expression and the frequency of cells expressing the cell-cycle-associated genes *CDK4*, *CDK6*, *CCND2*, and *TP53* were increased in SLE HSPCs within cluster 4 compared with healthy HSPCs although these changes did not reach statistical significance (**Figure 3F**).

**Figure 4:**
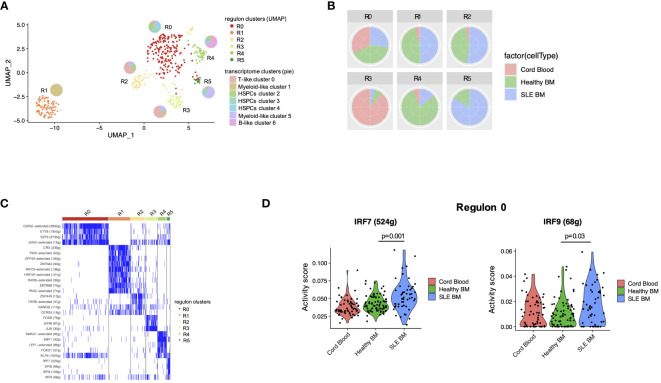
Transcription factor regulatory network analysis in HSPCs. **(A)** UMAP visualization of HSPC clustering based on regulons. Each pie chart shows the composition of each regulon cluster by cell type (defined based on single-cell gene expression). **(B)** Pie charts showing the composition of each regulon cluster by sample type. **(C)** Representative display of transcription factor regulon activity as binary outcome (blue: present, white = non-present) in each regulon. **(D)** Violin plots of IRF7 and IRF9 activity scores per sample type in regulon 0 (mainly HSPC clusters 2, 3, 4).

Transcription factors (TFs) are critical regulators of HSPC phenotype and lineage bias. In order to study the TFs that could act as regulators of the transcriptomic changes observed in patients with SLE, we performed TF regulatory network analysis of the single-cell data, which resulted in the identification of six clusters–regulons (**Figure 4A**). Regulon 0 included the HSPC clusters 2, 3, 4 (**Figure 4A**). Regarding the distribution of cells in the regulons, regulon 4 was enriched with healthy cells (mainly B-like cluster 6), which is characterized by the *SMAD1, EBF1, LEF1, FOXO1*, and *KLF6*-regulated networks. Regulon 5 was enriched with SLE cells (mainly myeloid-like cluster 5 cells), characterized by *SPIB* and the interferon-inducible *IRF7, IRF8, IRF9*-regulated networks (**Figures 4B, C**). Differential TF activity analysis between SLE and healthy controls within regulon 0, which includes the HPSC clusters, showed increased predicted activity of the TFs *IRF7* and *IRF9* as regulators of transcriptomic changes in SLE (p < 0.05, **Figure 4D**; **Supplementary Table 5**), further supporting that HSPCs in SLE are targeted by IFNs.

### 3.3 Differential alternative splicing in SLE HSPCs

**Figure 5:**
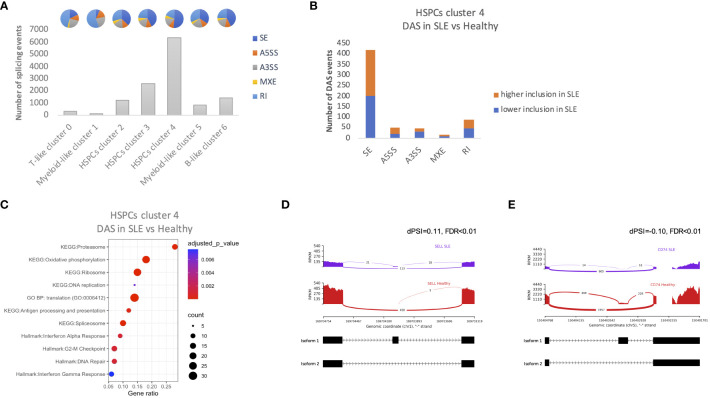
Alternative splicing events in HSPCs. **(A)** Number of total alternative splicing events in each cluster irrespective of disease or developmental stage. Each pie chart shows the composition of each cluster by alternative splicing event types (SE, skipped exon; RI, retained intron; MXE, mutually exclusive exons; A5SS, alternative 5′ splicing site; A3SS, alternative 3′ splicing site). **(B)** Number of differential alternative splicing (DAS) events in HSPC cluster 4 for each event type. Orange color represents the DAS events with higher inclusion level of exons in SLE and blue DAS events with lower inclusion level in SLE **(C)** Dot plot of EnrichR-enriched terms following differential alternative splicing analysis between SLE and healthy controls in HSPCs cluster 4. **(D, E)** Representative display of differential SELL and CD74 (IFN-related genes) exon usage in SLE versus healthy controls, respectively. The arcs connecting exons in the plot represent splice junctions, and their thickness provides a visual representation of the read coverage supporting those junctions. Differential splicing is measured in terms of the difference in the per cent spliced in dPSI. For example, the inclusion level of the splice site of *SELL* is 11% higher in SLE; therefore, the difference in PSI (SLE vs. healthy; dPSI) is 0.11.

Alternative splicing represents a vital component of the gene regulation process through the reduction of mRNA translation or the production of non-functional or malfunctional proteins. Previous studies have shown that SLE-related genes are subject to alternative splicing (28, 29). Therefore, we sought to identify whether alternative splicing events might affect hematopoiesis in patients with SLE. In total, we observed a larger number of splicing events in HSPC cluster 4 compared with other clusters (**Figure 5A**). Significant differential alternative splicing events between patients with SLE and healthy controls was observed in HSPC cluster 4 cells only with the most frequent type of event being skipped exon (**Figure 5B**). There were 383 genes affected by differential alternative splicing (DAS) in HSPC cluster 4, and these genes were enriched in pathways associated with cell proliferation, such as G2-M checkpoint, replication, translation and DNA repair, and interferon pathways (**Figure 5C**), providing supportive evidence that these mechanisms are dysregulated in SLE HSPCs. Particularly, IFN-related genes such as *SELL* and *CD74* were found to be affected by DAS giving rise to different proportions of two isoforms per gene in SLE when compared with healthy controls (**Figures 5D, E**).

## 4 Discussion

Hematopoietic progenitor cells are considered important players in the regulation of inflammation, being responsive to several inflammatory stimuli (7). Previous studies in preclinical mouse models of rheumatic diseases have shown that HSPCs are activated giving rise to inflammatory cells of the myeloid lineage (4–6, 10). Herein, we engaged single-cell RNA-seq to study, at the single-cell level, the molecular changes of human bone marrow-derived CD34+ progenitor cells in SLE. Based on the transcriptomic profile of this heterogeneous cell population (27), which includes both hematopoietic and myeloid progenitors, we were able to identify three clusters of cells with HSPC-like molecular profile. We observed that cells derived from two of these clusters, clusters 3 and 4, were enriched for genes associated with IFN signaling. Additionally, analysis for upstream regulators identified the interferon-dependent TFs IRF7 and IRF9 as possible TFs that drive the transcriptional modulation of HSPCs in SLE. Interestingly, there was a decrease in the number of cells in cluster 2 in SLE, which includes cells with increased quiescence, suggesting a transition of the HSPCs toward a proliferative state. Also, there was a decrease in the number of B-like cluster 6 cells in SLE and CB compared with healthy adults. In SLE, this is possibly due to the inflammation-driven myelopoiesis bias. On the other hand, in CB, due to the lack of exposure in environmental factors and inflammation, this is possibly due to the fact that these cells are more lineage primed based on their decreased stemness score.

Type I interferons (IFNs) are key players in steady state and autoimmunity. Interferon signaling is important for functional innate immunity, whereas various cell types produce interferons and express ISGs (interferon-stimulated genes) (30, 31). Terminally differentiated cells such as monocytes/macrophages, dendritic cells, and plasmacytoid cells produce IFNs in infection and autoimmunity. The type I-IFN pathway is genetically and mechanistically important for lupus pathogenesis (32–34). Transcriptomic analyses, both bulk and single-cell, have shown that high ISG expression represents a key signature for lupus pathogenesis (35–37). Interferon-targeted therapies (e.g., anifrolumab) (38) or cell-targeted therapies toward types producing type-I IFNs (e.g., belimumab) (39) have a constantly augmenting role in the armamentarium against SLE.

HSPCs are affected by IFN signaling, as shown in animal models (40–42). Chronic exposure to IFN-α is known to induce cell cycle entry and impair the stem cell activity in hematopoietic stem cells by inducing DNA damage (41), whereas dormant HSCs are protected from IFN-induced attrition by a circular RNA that binds and blocks the activity of the DNA sensor cGAS (43). To this direction, we show in the present study that SLE-dependent IFN signaling results in enhanced expression of cell cycle-related genes in cluster 4, a cluster that includes cells with enhanced proliferative potential. Except for the effect of type I interferons in cell-cycle entry, they can drive epigenetic changes in progenitor cells, which are reflected in their progeny. For instance, type I interferon signaling in progenitor cells in response to induction of trained immunity imprinted an inflammatory signature, which resulted in the generation of granulocytes with inflammatory and tumor suppressing properties (44). Based on this observation, we show herein that IFN signaling in SLE acts on bone marrow HSPCs, which could drive the generation of inflammatory cells of the myeloid lineage that can fuel disease activity and prime them to respond to secondary stimuli, contributing in the development of disease flares.

Even though peripheral destruction is the main cause of cytopenias in SLE, bone marrow failure has been also described. IFN signaling is the causal factor of aplastic anemia (AA), a prototypic autoimmune bone marrow failure disorder (45, 46). In AA, IFN-γ released in the bone marrow inflicts damage and attrition of CD34+ progenitor cells, causing pancytopenia (22, 46). Based on the effect of chronic IFN signaling in the suppression of HSC function, our data support a possible contribution of HSC dysfunction in SLE-associated cytopenias. Interestingly, altered alternative splicing events have been observed in HSPCs from patients with AA (22), further supporting a possible shared mechanism between the two disorders.

In addition to HSPCs, we identified a cluster of cells with myeloid transcriptomic features (myeloid-like cluster 5) that was significantly altered in patients with SLE. Specifically, within this cluster, genes associated with pathways associated with inflammation, including IFN and TNF signaling, the cell cycle-related pathway G2/M transition and hypoxia, and reactive oxygen species pathway, were upregulated in SLE. Previous studies have shown that there is a myeloid cell signature in bone marrow progenitor cells in SLE (4), whereas neutrophils and monocytes are critical effector cell populations in the pathogenesis of severe disease complications, such as nephritis (32). In this direction, in addition to HSPCs, we show that progenitor cells with a myeloid bias based on the transcriptomic signature are affected by IFN, whereas other clusters of cells such as cells with a T- and B-like signature are unaffected.

A fundamental question is whether the IFN-related signature in HSPCs acts as the initiator of disease phenotype, or it is a secondary effect that further amplifies the cascade of events that characterize SLE-dependent inflammation. This study cannot be addressed by our experimental setting, since BM samples from asymptomatic subjects that will develop SLE in the future are needed to distinguish whether HSPCs are the starting point of the inflammatory process in SLE, which makes this type of experimental process not possible.

Taken together, single-cell analysis of BM cells from patients with SLE demonstrated that interferon signaling, a major player in disease pathogenesis, affects early hematopoietic progenitors. Early imprinting of the interferon signature in HSPCs is likely to affect their progeny downstream, promoting the initiation and progression of the disease. Together, these data suggest that the fundamental aberrancies in SLE could be traced back to the HSPCs. Whether novel therapies that target IFNs can reverse the imprinted inflammatory signature in HSPCs and to what extent this reversal can result in the modulation of the function of the generated immune cells is to be shown.

## Data availability statement

The dataset has been deposited at the EGA controlled-access repository (EGAS00001007317).

## Ethics statement

The studies involving humans were approved by Attikon University Hospital Research Ethic Committee (Athens, Greece, protocol 10/22-6-2017). The studies were conducted in accordance with the local legislation and institutional requirements. The participants provided their written informed consent to participate in this study.

## Author contributions

AF: Writing – original draft, Writing – review & editing, Formal analysis, Investigation, Methodology, Validation. IM: Writing – original draft, Writing – review & editing, Investigation, Methodology. CL: Methodology, Writing – review & editing. MG: Methodology, Writing – review & editing. AB: Investigation, Writing – review & editing. GS: Methodology, Writing – review & editing. SG: Resources, Writing – review & editing. VK: Resources, Writing – review & editing. EA: Methodology, Writing – review & editing. IK: Investigation, Methodology, Writing – original draft, Writing – review & editing. DB: Conceptualization, Funding acquisition, Resources, Supervision, Writing – original draft, Writing – review & editing.
