# Supplementary material for: Single-cell transcriptomic analysis of hematopoietic progenitor cells from patients with systemic lupus erythematosus reveals interferon-inducible reprogramming in early progenitors
Source: Front Immunol. 2024 May 8;15:1383358. doi: 10.3389/fimmu.2024.1383358 (PMC11109438; doi:10.3389/fimmu.2024.1383358)
Supplement: Supplementary file 1 [file DataSheet_1.docx]

**Supplementary figures**

**
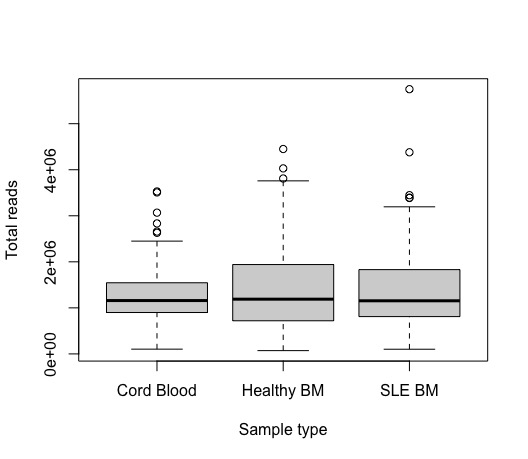
**

**Supplementary Figure1.** Box plot showing the number of total sequenced reads per sample type.

**
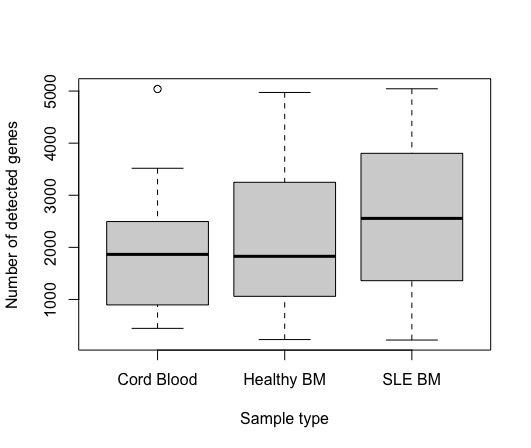
**

**Supplementary Figure 2.** Box plot showing the number of detected genes per sample type.


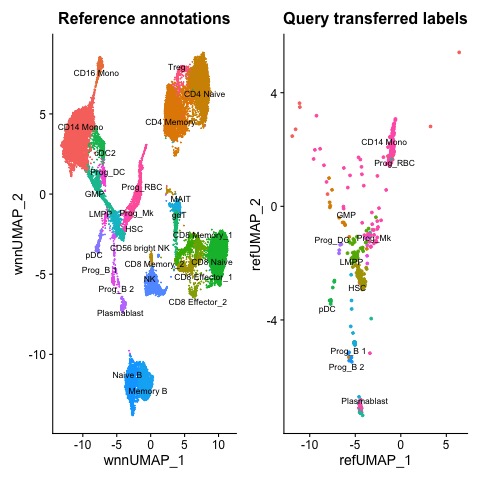


**Supplementary Figure 3.** UMAP plot showing our single cell RNA sequencing dataset (on the right) projected onto a reference dataset (on the left). The reference dataset was derived from human bone marrow mononuclear cells (BMMCs) which is available through Seurat R package.


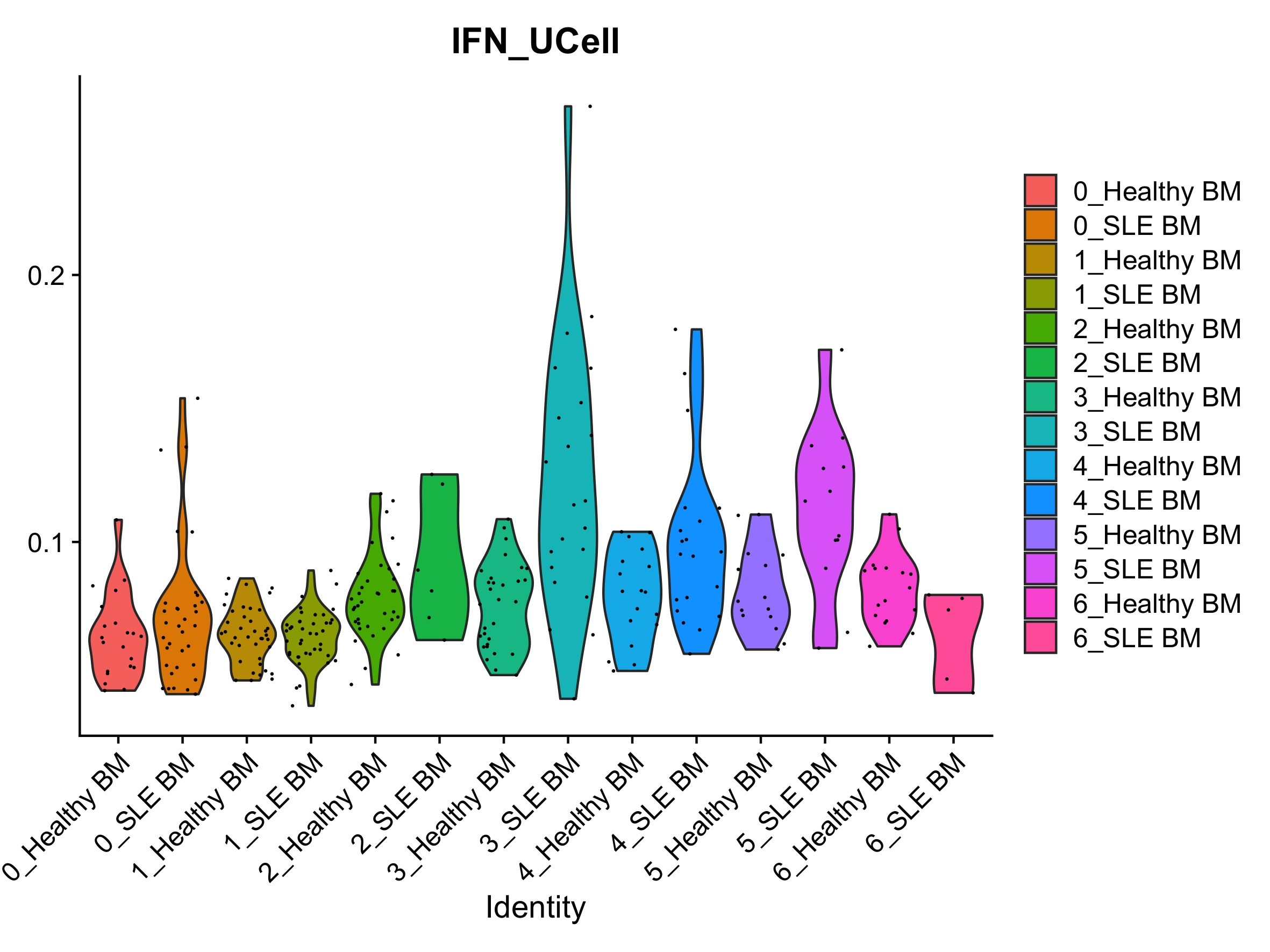


**Supplementary Figure 4.** Violin plots of IFN signature score of each cluster of SLE and healthy controls.
